# An examination of the factorial and convergent validity of four measures of conspiracist ideation, with recommendations for researchers

**DOI:** 10.1371/journal.pone.0172617

**Published:** 2017-02-23

**Authors:** Viren Swami, David Barron, Laura Weis, Martin Voracek, Stefan Stieger, Adrian Furnham

**Affiliations:** 1 Department of Psychology, Anglia Ruskin University, Cambridge, Cambridgeshire, United Kingdom; 2 Department of Psychology, HELP University College, Kuala Lumpur, Malaysia; 3 Department of Psychology, University of Westminster, London, United Kingdom; 4 Department of Clinical, Educational, and Health Psychology, University College London, London, United Kingdom; 5 Department of Basic Psychological Research and Research Methods, School of Psychology, University of Vienna, Vienna, Austria; 6 Research Methods, Assessment, and iScience, Department of Psychology, University of Konstanz, Konstanz, Germany; Universiteit van Amsterdam, NETHERLANDS

## Abstract

A number scales have been developed to measure conspiracist ideation, but little attention has been paid to the factorial validity of these scales. We reassessed the psychometric properties of four widely-used scales, namely the Belief in Conspiracy Theories Inventory (BCTI), the Conspiracy Mentality Questionnaire (CMQ), the Generic Conspiracist Beliefs Scale (GCBS), and the One-Item Conspiracy Measure (OICM). Eight-hundred-and-three U.S. adults completed all measures, along with measures of endorsement of 9/11 and anti-vaccination conspiracy theories. Through both exploratory and confirmatory factor analysis, we found that only the BCTI had acceptable factorial validity. We failed to confirm the factor structures of the CMQ and the GBCS, suggesting these measures had poor factorial validity. Indices of convergent validity were acceptable for the BCTI, but weaker for the other measures. Based on these findings, we provide suggestions for the future refinement in the measurement of conspiracist ideation.

## Introduction

In tandem with growing scholarly interest in the psychology of conspiracy theories [1], researchers have developed a range of different scales to measure individual differences in conspiracist ideation, which we define broadly here as a tendency to endorse conspiracy theories or engage in conspiracist thinking. With few exceptions, however, most of these newly developed scales have not been subjected to thorough investigations of their psychometric properties. In particular, little attention has been paid to the factorial and convergent validity, and internal consistency, of these scales, which is concerning because scholars may be inadvertently introducing a degree of bias into their studies [2].

In this article, we review current approaches to measuring individual differences in conspiracist ideation. In brief, two different approaches are evident in the literature: (a) measuring conspiracist ideation in terms of endorsement of a range of real-world conspiracy theories, and; (b) measuring conspiracist ideation in generic terms without reference to real-world conspiracy theories. We review the different scales that have been developed in alignment with these approaches and highlight their measurement-related deficiencies, particularly in terms of factorial validity. In addition, we report on a new dataset from U.S. participants, via which we re-examine the psychometric properties of four measures of conspiracist ideation. This allows for the most comprehensive assessment of such scales to date and allows us to make recommendations for their future use.

### Endorsement of a range of conspiracy theories

Most early scales that were developed to measure conspiracist ideation relied on a similar underlying principle: that by presenting participants with a range of real-world conspiracy theories (e.g., the moon landings were faked), it would be possible to obtain an overall measure of conspiracist ideation (or, more accurately, global endorsement of conspiracy theories). A number of such scales have been developed (see Table 1), including the Belief in Specific Conspiracies Scale [3], the Conspiracy Theory Belief Scale [4], the Composite Conspiracy Beliefs Scales [5], and the Belief in Conspiracy Theories Inventory [6]. These scales vary widely in terms of the information provided about scale development, item construction and content, number of items, and internal consistency. Importantly, there has been a tendency for scholars to treat these scales as factorially unidimensional (i.e, by computing total scores) in the absence of analyses of their factor structures [3–5] or to treat the items individually [7].

**Table 1:** Scales that measure endorsement of a range of conspiracy theories.

Measure	Reference	Language	*N*	No. of items	Anchors	Factorial validity	Cronbach α	Test-retest reliability	Convergent validity
Belief in Specific Conspiracies Scale	[3]	English	156 US university students	22	1 = *Strongly disagree*, 7 = *Strongly agree*	Not examined	.89	Not examined	None
Belief in Conspiracy Theories Scale	[47], Study 1	English	30 UK undergraduates	8	1 = *Strongly disagree*, 5 = *Strongly agree*	Not examined	Not reported	Not examined	Correlation with attribution of novel event to conspiracy not significant, *r* < .01
	[47], Study 2	English	86 UK undergraduates	8	1 = *Strongly disagree*, 5 = *Strongly agree*	Not examined	Not reported	Not examined	None
Composite Conspiracy Beliefs Scale	[5], Study 2a	Dutch	1,010 Dutch adults, representative of the Netherlands	6	1 = *Highly probably*, 7 = *Highly improbable*	Not examined	.80	Not examined	None
	[5], Study 2b	Dutch	1,297 Dutch adults, representative of the Netherlands	6	1 = *Highly probably*, 7 = *Highly improbable*	Not examined	.82	Not examined	None
	[5], Study 3	Dutch	268 Dutch adults from an online sample	9	1 = *Highly probably*, 7 = *Highly improbable*	Not examined	.86	Not examined	None
	[7], Study 2	Dutch	1256 US adults from online samples	5	1 = *Definitely false*, 5 = *Definitely true*	Not examined	Items treated individually	Not examined	None
Conspiracy Theory Beliefs Scale	[4], Study 1	English	189 UK undergraduates	17	1 = *Never under any circumstances*, 7 = *Probably yes*	Not examined	.82	Not examined	None
	[4], Study 2	English	60 UK undergraduates	17	1 = *Never under any circumstances*, 7 = *Probably yes*	Not examined	Not reported	Not examined	None
	[8], Study 1	English	137 UK undergraduates	17	1 = *Strongly disagree*, 7 = *Strongly agree*	EFA revealed two factors measuring generic conspiracy theories and climate change conspiracy theories	Total scale = .78; subscales not reported	Not examined	None
	[12], Study 1	English	202 online adults, location unspecified	7	1 = *Strongly disagree*, 7 = *Strongly agree*	Not examined	.82	Not examined	None
	[12], Study 2	English	328 online adults, location unspecified	17	1 = *Strongly disagree*, 7 = *Strongly agree*	Not examined	.87	Not examined	None
	[11], Study 1	English	91 UK adults from the community	Not reported (17 presumed)	1 = *Strongly disagree*, 7 = *Strongly agree*	Not examined	.96	Not examined	None
	[13], Study 1	English	186 UK university students	12	1 = *Extremely unlikely*, 7 = *Extremely likely*	Not examined	.90	Not examined	None
Belief in Conspiracy Theories Inventory	[6]	English	257 adults representative of UK population	15	1 = *Completely false*, 9 = *Completely true*	Principal axis EFA: 14 items load onto primary factor, 1 item dropped	.86	Not examined	Measure of 9/11 conspiracist beliefs, *r* = .55
	[14]	English	914 UK adults from the community	14	1 = *Completely false*, 9 = *Completely true*	Not examined	.89	Not examined	Measure of belief in conspiracy theories about the disappearance of Amelia Earhart, *r* = .12
	[15], Study 1	English	817 UK adults from the community	15 (14 from parent study plus on item about 9/11 conspiracy theory)	1 = *Completely false*, 9 = *Completely true*	Not examined	.90	Not examined	Measure of 7/7 bombings conspiracist beliefs, *r* = .75
	[16]	English	259 US adults from online sample	15	1 = *Completely false*, 9 = *Completely true*	Not examined	.93	Not examined	None
	[48], Study 1	English	990 UK adults from the community	15	1 = *Completely false*, 9 = *Completely true*	Not examined	.91	Not examined	None
	[48], Study 2	English	112 UK undergraduates	15	1 = *Completely false*, 9 = *Completely true*	Not examined	.87-.89	Not examined	None
	[48], Study 3	English	189 UK undergraduates	15	1 = *Completely false*, 9 = *Completely true*	Not examined	.88-.90	Not examined	None
	[49]	English	420 US adults from online sample	15	1 = *Completely false*, 9 = *Completely true*	Not examined	.92	Not examined	None
	[50]	English	447 adults mainly from UK and US, from online sample	15	1 = *Completely false*, 9 = *Completely true*	Not examined	.92	Not examined	None
	[15], Study 2)	German	281 central European adults from the community	15	1 = *Completely false*, 9 = *Completely true*	Not examined	.87	Not examined	Measure of belief in a fictitious conspiracy theory, *r* = .55
	[51]	German	281 and 273 central European adults from the community	15	1 = *Completely false*, 9 = *Completely true*	Not examined	.87	Not examined	Measure of belief in conspiracy theory about Natascha Kampusch, *r*s = .56-.59
	[52], Study 1	German	192 central European adults from the community	15	1 = *Completely false*, 9 = *Completely true*	Not examined	.88	Not examined	Measure of belief in moon landings conspiracy theories, *r* = .59
	[52], Study 2	German	392 central European adults from the community	15	1 = *Completely false*, 9 = *Completely true*	Not examined	.86	Not examined	Measure of belief in moon landings conspiracy theories, *r* = .54
	[53]	German	494 central European adults from the community	15	1 = *Completely false*, 9 = *Completely true*	Not examined	.90	Not examined	None
	[54], Study 1	English	107 Australian adults (unspecified)	15	1 = *Completely false*, 9 = *Completely true*	Not examined	.93	Not examined	Measure of belief in 9/11 conspiracy theories, *r* = .77
	[54], Study 2	English	121 Australian adults (unspecified)	15	1 = *Completely false*, 9 = *Completely true*	Not examined	.94	Not examined	Measure of belief in fictitious conspiracy theory, *r* = .78; Measure of ‘true’ conspiracy theories, *r* = .75; GCB, *r* = 83; CMQ, *r* = .62
	[17], Study 1	Malay	368 Malay adults from the community in Malaysia	15	1 = *Completely false*, 9 = *Completely true*	Not examined	.90	Not examined	Measure of belief in Jewish conspiracy theory, *r* = .22
	[17], Study 2	Malay	314 Malay adults from the community in Malaysia	15	1 = *Completely false*, 9 = *Completely true*	Not examined	.88	Not examined	Measure of belief in Jewish conspiracy theory, *r* = .17
	[18], Study 1	French	152 French Masters students	10 selected to be recognisable to French audience	1 = *Completely false*, 9 = *Completely true*	Not examined	.83	Not examined	Single-item conspiracy theory, *r* = .50; GCB, *r* = .66; CMQ, *r* = .38
	[18], Study 2	English	292 US adults from online sample	15	1 = *Completely false*, 9 = *Completely true*	Not examined	.85	Not examined	Single-item conspiracy theory, *r* = .66, GCB, *r* = .83; CMQ, *r* = .65

To date, only two of these measures have been subjected to factor analysis. One study [8] submitted the 17 items of the Conspiracy Theory Belief Scale to exploratory factor analysis (EFA) and extracted two distinct factors relating to generic conspiracy theories and climate change conspiracy theories. However, it is not apparent that the study had a sufficiently large size (*N* = 138) by conservative participant-to-item standards (i.e., a participant-to-item ratio of 10:1) [9] to conduct EFA. Moreover, the authors [8] elected to compute a total score (Cronbach α = .78), arguing that item inter-correlations were high. This is problematic because item inter-correlations and high internal consistencies may still mask underlying latent factors [10] and, in any event, the internal consistency of the total score was below what has described as acceptable for novel measures (i.e., a internal consistency coefficient of .80) [9]. Other studies using this measure have likewise computed total scores and have reported higher internal consistency coefficients [11–13], but have neglected to examine the scale’s factor structure. At least one study [12] has also used a truncated version of this scale in the absence of an examination of the scale’s dimensionality.

A different measure is the Belief in Conspiracy Theory Inventory (BCTI) [6]. In the parent study, the authors [6] subjected a pool of 15 items to EFA and reported that all but one of the items loaded onto a primary factor. They, therefore, computed a total BCTI score as the mean of the 14 remaining items, a method that has been used in one other study [14]. In a later study [15], an additional item was added to the list of 14 items and a total score was computed, but the authors neglected to report on the factorial validity of this adapted measure. Subsequent studies have mostly used the 15-item version of the BCTI and, although acceptable internal consistency coefficients have been reported [16], none of these studies have re-examined the factorial validity of the BCTI. In addition, the measure has been translated into German [15] and Malay [17], but in both instances the scale translators have not reported on the dimensionality of the measure. A shorter, 10-item version of the scale has also been translated into French [18], but again a total score was computed in the absence of evidence of a one-factor structure.

In addition to the lack of evidence of factorial validity, these scales also suffer from a number of additional problems. As noted in Table 1, very few of these studies have provided estimates of convergent validity for the scales being used. Response options have also varied between studies for some scales and sample sizes in the studies have varied widely. Perhaps the most problematic aspect of these scales, however, relates to their construct validity. It is not clear to what extent these scales measure anything other than belief in a set of real-world conspiracy theories. Even if we accept that they measure individual differences in conspiracist ideation, such scales may be impractical, requiring constant updating to reflect changes in the popularity of particular conspiracy theories or to reflect local knowledge of conspiracy theories.

### Measures of generic conspiracist ideation

Some scholars have developed measures of generic conspiracist ideation that do not make reference to specific conspiracy theories. Such generic conspiracist ideation, would in turn be expected to be positively associated with endorsement of specific conspiracy theories. There are a number of such scales (see Table 2), including the Conspiracy Theory Questionnaire [19], a subscale of the Epistemically Unwarranted Beliefs Scale [20], the Conspiracy Mentality Questionnaire (CMQ) [21], and the Generic Conspiracist Belief Scale (GCBS) [22]. Notably, the former two scales have not been subjected to factor analysis and one-factor structures have been assumed in the absence of empirical evidence in their favour. The latter two scales have been subjected to EFA and confirmatory factor analysis (CFA), but likewise suffer from a number of limitations.

**Table 2:** Scales that measure generic conspiracist ideation.

Measure	Reference	Language	*N*	No. of items	Anchors	Factorial validity	Cronbach α	Test-retest reliability	Convergent validity
Conspiracy Theory Questionnaire	[19]	English	120 UK university students	38	1 = *Extremely unlikely*, 9 = *Certainly*	Not examined	.96	Not examined	None
	[55]	English	223 mixed sample	38	1 = *Certainly not*, 11 = *Certainly*	Not examined	.72	Not examined	Measure of generic conspiracist beliefs, *r* = .56; endorsement of alternative explanations for historical events, *r* = .63
Conspiracy Mentality Questionnaire	[23], Study 1a	English	497 adults from online sample (location not reported)	12	Not reported	CFA showed that a one-factor model had adequate fit	.90	Not examined	None
	[23], Study 1b	German	133 adults (recruitment not specified)	12	Not reported	Not examined	Not reported	15-day interval, *r* = .88	None
	[23], Study 1c	German	63 adults (recruitment not specified)	12	Not reported	Not examined	Not reported	1-year interval, *r* = .67	None
	[23], Study 2	German	294 adults from online sample	12	Not reported	Not examined	.89	Not examined	None
	[23], Study 3	German	280 German university students	12	Not reported	Not examined	.89	Not examined	None
	[23], Study 4	German	280 German university students	12	Not reported	Not examined	.89	Not examined	None
	[23], Study 5	German	1852 German adults from online sample	12	Not reported	Not examined	.89	Not examined	None
	[21], Study 1a	German, English, and Turkish	7766 online adults from Germany, UK, US, Ireland, and Turkey	5	0% = *Certainly not*, 100% = *Certain*	EFA, one-factor model extracted; multi-group CFA showed adequate fit across groups	.72 (Turkish), .84 (English and German)	Not examined	Endorsement of 33 conspiracy theories, *r*s = .37-.76
	[21], Study 1b	German	133 German university students	5	0% = *Certainly not*, 100% = *Certain*	Not examined	.77-.82	15-day interval, *r* = .84	None
	[21], Study 2	English	120 UK university students	5	0% = *Certainly not*, 100% = *Certain*	Not examined	.85	Not examined	Endorsement of 33 conspiracy theories, *r*s = .30-.81
	[21], Study 3	English	76 UK adults from the community	5	0% = *Certainly not*, 100% = *Certain*	Not examined	.73	Not examined	Endorsement of 33 conspiracy theories, *r*s = .20-.69
	[21], Study 4	German	274 German university students	5	0% = *Certainly not*, 100% = *Certain*	Not examined	.78	Not examined	Novel conspiracy mentality questionnaire, *r* = .82; Endorsement of 33 conspiracy theories, *r*s = .32-.68
	[54], Study 2	English	121 Australian adults (unspecified)	5	0% = *Certainly not*, 100% = *Certain*	Not examined	.84	Not examined	Measure of belief in fictitious conspiracy theory, *r* = .61; Measure of ‘true’ conspiracy theories, *r* = .51; BCTI, *r* = 62; GCB, *r* = .65
	[18], Study 1	French	152 French Masters students	5	0% = *Certainly not*, 100% = *Certain*	Not examined	.79	Not examined	Single-item conspiracy theory, *r* = .41; BCTI-10, *r* = .38; GCB, *r* = .55
	[18], Study 2	English	292 US adults from online sample	5	0% = *Certainly not*, 100% = *Certain*	Not examined	.84	Not examined	Single-item conspiracy theory, *r* = .70, BCTI, *r* = .65; GCB, *r* = .75
Epistemically Unwarranted Beliefs Scale	[20]	English	480 US undergraduates	10	1 = *Strongly disagree*, 5 = *Strongly agree*	Not examined	.67	Not examined	None
Generic Conspiracist Belief Scale	[22], Study 1	English	489 mixed US and UK undergraduates	Originally 75 (59 following EFA)	1 = *Definitely not true*, 5 = Definitely true	EFA on 59 positively worded items; 5 factors extracted	Subscales .87-.95; Total score not reported	Not examined	None
	[22], Study 2	English	225 UK undergraduates	15 selected to be representative of 5 factors in Study 1	1 = *Definitely not true*, 5 = Definitely true	CFA of 5-factor model showed adequate fit; 5-factor model had better fit than 1-factor model	Total score = .93; subscales not reported	5-week interval, *r* = .89	BCTI (*n* = 202), *r* = .82; measure of 9/11 conspiracy theories (*n* = 206), *r* = .75; 7/7 conspiracy theories (*n* = 205), *r* = .67; fictitious conspiracy theory (*n* = 209), *r* = .61
	[22], Study 3	English	208 UK adults from Psychology of Paranormal e-list	15	1 = *Definitely not true*, 5 = Definitely true	Not examined	Total score = .95; subscales not reported	Not examined	BCTI, *r* = .86
	[22], Study 4	English	194 mixed US and UK adults from online sample	15	1 = *Definitely not true*, 5 = Definitely true	Not examined	Total score = .95; subscales not reported	Not examined	None
	[48], Study 4	English	140 UK adults from the community	15	1 = *Definitely not true*, 5 = Definitely true	Not examined	Total score = .91; subscales not reported	Not examined	Measure of 7/7 bombings conspiracist beliefs, *r* not reported
	[54], Study 2	English	121 Australian adults (unspecified)	15	1 = *Definitely not true*, 5 = Definitely true	Not examined	Total score = .95; subscales not reported	Not examined	Measure of belief in fictitious conspiracy theory, *r* = .68; Measure of ‘true’ conspiracy theories, *r* = .60; BCTI, *r* = 83; CMQ, *r* = .65
	[18], Study 1	French	152 French Masters students	15	1 = *Definitely not true*, 5 = Definitely true	Not examined	Total score = .85; subscales not reported	Not examined	Single-item conspiracy theory, *r* = .50; BCTI-10, *r* = .66; CMQ, *r* = .55
	[18], Study 2	English	292 US adults from online sample	15	1 = *Definitely not true*, 5 = Definitely true	Not examined	Total score = .94; subscales not reported	Not examined	Single-item conspiracy theory, *r* = .72, BCTI, *r* = .83; CMQ, *r* = .75
	[11]	English	95 UK undergraduates	15	1 = *Definitely not true*, 5 = Definitely true	Not examined	Total score = .88; subscales not reported	Not examined	None
	[56], Study 1	English	84 UK undergraduates	15	1 = *Definitely not true*, 5 = Definitely true	Not examined	Total score = .90; subscales not reported	Not examined	None
	[56], Study 2	English	102 UK Psychology undergraduates	15	1 = *Definitely not true*, 5 = Definitely true	Not examined	Total score = .88; subscales not reported	Not examined	None
	[56], Study 3	English	84 Psychology students	15	1 = *Definitely not true*, 5 = Definitely true	Not examined	Total score = .92; subscales not reported	Not examined	None
	[57]	Not specified (English presumed)	150 adults from multiple countries	15	1 = *Definitely not true*, 5 = Definitely true	Not examined	Total score = .97; subscales not reported	Not examined	None
	[58]	Not specified (English presumed)	209 Canadian undergraduates	15	1 = *Definitely not true*, 5 = Definitely true	Not examined	Total score = .92; subscales not reported	Not examined	None
	[24], Study 1	French	107 French Psychology undergraduates	15	1 = *Definitely not true*, 5 = Definitely true	Not examined	Total score = .85; subscales not reported	Not examined	Measure of ‘classical’ conspiracy theories, *r* = .46
	[24], Study 2	French	123 French Psychology undergraduates	15	1 = *Definitely not true*, 5 = Definitely true	Not examined	Total score = .82; subscales not reported	Not examined	Measure of ‘classical’ conspiracy theories, *r* = .68
	[24], Study 3	French	213 French adults from online sample	15	1 = *Definitely not true*, 5 = Definitely true	Not examined	Total score = .88; subscales not reported	Not examined	Measure of ‘classical’ conspiracy theories, *r* = .63
	[59], Study 1	English	150 US adults from online sample	15, converted to the form of questions	1 = *Not at all likely*, 5 = *Extremely likely*	Not examined	Total score = .95; subscales not reported	Not examined	Endorsement of 5 US historical conspiracy theories, *r* = .75
	[59], Study 2	English	802 US adults from online sample	15, converted to the form of questions	1 = *Not at all likely*, 5 = *Extremely likely*	Not examined	Total score = .93; subscales not reported	Not examined	None
	[60], Study 1	English	202 US adults from online sample	15	1 = *Definitely not true*, 5 = Definitely true	Not examined	Total score = .93; subscales not reported	Not examined	None
	[60], Study 1	English	269 US adults from online sample	15	1 = *Definitely not true*, 5 = Definitely true	Not examined	Total score = .91; subscales not reported	Not examined	None
	[25]	Macedonian	160 Macedonian adults from an online sample	15	1 = *Definitely not true*, 5 = Definitely true	Not examined	Total score = .91; subscales not reported	Not examined	None
	[61]	English	202 US adults from online sample	15	1 = *Definitely not true*, 5 = Definitely true	Not examined	Total score = .95; subscales not reported	Not examined	None

To take the CMQ first, two different versions of this scale appear to exist in the literature: a 12-item version [23] and a 5-item version [21]. The first of these has been subjected to CFA, which showed a one-factor solution to have acceptable fit, but CFA is an inappropriate analytic strategy for a novel scale. CFA indicates whether a hypothesised model has adequate fit, but tells scholars little about whether there may be alternative, better-fitting models. In addition, the authors [23] also appear to have neglected to report on the response option for this 12-item measure. On the other hand, the 5-item version has been subjected to EFA [21], with a one-factor solution extracted. Additionally, multi-group CFA showed that the one-dimensional model had adequate fit in German- and English-speaking samples, but indices for a Turkish-speaking sample were problematic. Even so, the 5-item CMQ may be difficult-to-understand and some studies have reported internal consistency coefficients below an acceptable cut-off [9] (see Table 2).

Further problems with the CMQ include insufficient information about its construction and original item pool, as well as concerns related to its construct validity (i.e., it is not entirely clear that all items in the scale reflect conspiracist ideation, which may explain its low internal consistency in some studies). More specifically, of the five items included in the CMQ, only two (items #4 and #5 may directly assess conspiracist ideation as it is currently conceived. Item #3 is almost certainly factual, but may not necessarily require an underlying conspiracist belief. Items #1 and #2 likewise could be construed as statements of fact, without any underlying conspiracist motive.

The GCBS is perhaps the most widely used measure of generic conspiracist ideation. In the parent study, the authors [22] reported on the development of a pool of 75 initial items, which was reduced to 59 follow exclusion of negatively-worded items. Based on an EFA of the remaining items, five factors with acceptable internal consistencies were extracted. In a second study [22], the authors selected 15 “representative” items and reported that CFA showed a five-factor model to have acceptable fit and better fit than a one-factor model with all 15 items. Even so, they and all subsequent studies using the GCBS have shown a preference to work with total scores. Two further problems limit the validity of the GCBS. First, the authors did not have a sufficiently large sample size to conduct EFA in the parent study; further examinations of the scale’s factor structure were also conducted with small samples with suspect generalisability (see Table 2). Second, the GCBS has been translated into French [24] and Macedonian [25], but factorial validity in these new cultural contexts has not been investigated.

### One-Item Conspiracy Measure

To the above list of measures, one study [18] recently added a one-item measure of conspiracist ideation. Although this measure was designed for use when scholars are pressed for time, and although it is not possible to examine the factor structure or report on the internal consistency of this measure, the authors reported that the one-item measure had adequate patterns of convergent validity (see Table 3) and acceptable test-retest reliability after 14 days (*r* = .75). Given the issues discussed above concerning dimensionality of conspiracist ideation, it is not immediately apparent to what extent a one-item measure offers practical utility over other measures that are already relatively brief. Moreover, in some cases (see Table 3), convergent validity estimates that have been reported for the scale have been moderate at best, raising questions about the extent to which it truly captures individual differences in conspiracist ideation.

**Table 3:** One-Item Conspiracy Measure.

Measure	Reference	Language	*N*	No. of items	Anchors	Factorial validity	Cronbach α	Test-retest reliability	Convergent validity
One-Item Conspiracy Measure	Lantian, Muller, Nurra, & Douglas (2016, Study 1)	French	152 French Masters students	1	1 = *Completely false*, 9 = *Completely true*	Not possible	Not possible	Not examined	GCB, *r* = .50; BCTI-10, *r* = .50; CMQ, *r* = .41
	Lantian, Muller, Nurra, & Douglas (2016, Study 2)	English	292 US adults from online sample	1	1 = *Completely false*, 9 = *Completely true*	Not possible	Not possible	Not examined	GCB, *r* = .72; BCTI, *r* = .66; CMQ, *r* = .70
	Lantian, Muller, Nurra, & Douglas (2016, Study 3)	French	73 French Psychology undergraduates	1	1 = *Completely false*, 9 = *Completely true*	Not possible	Not possible	14-day interval, *r* = .75	

### One-dimensional or multi-dimensional?

The issues discussed above should give pause to scholars who want to operationalise and measure individual differences in conspiracist ideation. While there has been a proliferation of a range of conspiracist ideation scales, measurement issues have not been paid adequate attention. This has resulted in a number of scales with uncertain psychometric properties. Where factor structures have been examined, it is not immediately clear that scholars have applied basic guidelines for conducting factor analyses, explored the possibility of alternative models, critically appraised the decision(s) to utilise total scores, or re-examined factorial validity when the scales were used in new linguistic or cultural groups. In other instances, scholars have not fully reported on scale construction, making it difficult for scholars interested in replication efforts. These are all issues that have the potential to substantially hamper efforts to measure conspiracist ideation.

In addition, there remains some confusion in the theoretical foundations that have led to the construction of the afore-mentioned scales, particularly as to whether conspiracist ideation can be considered to be a one-dimensional or multi-dimensional construct. In terms of scales that measure endorsement of a range of conspiracy theories, the available evidence would seem to suggest that such measures should be one-dimensional. This is based on the finding that belief in conspiracy theories tends to be “monological” [6, 26]. That is, belief in one conspiracy theory tends to make assimilation of other conspiracy theories more likely; as such, when participants are asked to complete measures that tap endorsement of multiple conspiracy theories, one should expect a monological belief system in which belief in a range of conspiracy theories are inter-correlated.

The dimensionality of conspiracist ideation, on the other hand, remains an open question. Although it is possible that conspiracist ideation is multi-dimensional, consisting of discrete beliefs about multiple conspiratorial acts [22], in practice most scholars have assumed that conspiracist ideation should be considered an internally coherent and one-dimensional trait. This is reflected in the use of total scores for the GCBS, as well as a one-dimensional factor structure of the CMQ. Likewise, the one-item measure of conspiracist ideation assumes that the construct can be reduced to a single dimension. Such assumptions appear to be predicated on the idea that conspiracist ideation can be considered to be a latent personality trait, akin to paranormal beliefs for example. While such an assumption seems intuitively plausible, it needs to be rigorously tested before firm conclusions can be drawn.

### The present study

Additional research is clearly needed to increase researchers’ understanding of, and confidence in, measures used to assess conspiracist ideation. Here, we sought to cast fresh light on some of these measurement issues (i.e., factorial validity, convergent validity, and internal consistency) *vis-à-vis* the BCTI, the GCBS, the CMQ, and the one-item conspiracy measure. The three former measures were selected because they are currently the most widely-used measures in the literature and also because their parent studies have reported on the factorial validity of the measures. In addition, we included the one-item measure because it is the most recently validated. We elected to omit the Conspiracy Theory Belief Scale for a number of reasons: there appears to be a good deal of item overlap between items in this measure and the BCTI, and responses scales for this measure have varied across studies (see Table 1). In addition, unlike the BCTI (its most closely comparable scale), the GCBS, and the CMQ, the Conspiracy Theory Belief Scale has been used only relatively infrequently in the literature.

In terms of factorial validity, we gathered data from a large U.S. sample of adults, which allowed us to first examine the factor structures of these measures using EFA (to suggest an acceptable, best-fitting structure) and then use CFA in a randomly-selected split-half of the sample (to cross-validate the models). In terms of convergent validity, in addition to assessing scale inter-correlations, we also included a measure of belief in a 9/11 conspiracy theory (i.e., the belief that the September 11, 2011, terrorist attacks were orchestrated or allowed to occur by the U.S. government) and an anti-vaccination conspiracy theory (i.e., the belief that vaccinations do not serve their intended purpose). Finally, we also re-assessed internal consistency coefficients of the four target scales using Nunnally’s [9] widely-cited, but often incorrectly interpreted, criterion.

## Materials and methods

### Ethics statement

This study was conducted in accordance with the principles expressed in the Declaration of Helsinki and was approved by the ethics committee of the Department of Psychology, University of Westminster (application number: VRE1516-1352). All participants provided written informed consent.

### Procedures and participants

The study was approved by the relevant university ethics committee. Data were collected via Amazon’s Mechanical Turk (MTurk) website on May 6–7, 2016. MTurk is a crowdsourcing Internet marketplace that allows individuals and businesses (Requesters) to ask “workers” to complete tasks for payment. MTurk samples are increasingly being used in psychological studies, as it provides a source of high-quality data, and have been reported to be more demographically-diverse than standard Internet samples [27]. The project was advertised as a study on “political opinions and attitudes” and included an estimated duration and compensation. The questionnaire was advertised to MTurk workers who achieved a > 98% approval rate and completed at least 1,000 hits. We limited participation to MTurk workers from the U.S. so as to achieve a relatively homogeneous sample in terms of cultural identity. After providing informed consent, participants were directed to the measures described below, which were presented in an anonymous form and in random order via the randomisation function with Qualtrics, which hosted the survey. In exchange for completing the survey, participants were paid $0.75. Forty-six participants with large amounts of missing data (i.e., missing more than 10% of the total data across all measures) [28] were excluded from the dataset prior to analyses. For all remaining participants, missing data (< 0.2% of total dataset) were completely at random (based on Little’s MCAR analyses), so we used the mean replacement technique to estimate missing values. All participants received debriefing information at the end of the survey.

The final sample consisted of 448 women and 355 men, ranging in age from 18 to 70 years (*M* = 37.07, *SD* = 11.94). The majority of participants self-reported as White (84.4%), while 6.1% were of African American ancestry, 5.6% of Asian ancestry, and 3.8% as some other ethnic background. In terms of educational qualifications, 27.3% had completed high school, 4.0% were still in full-time education, 49.7% had an undergraduate degree, 15.6% had a postgraduate degree, and the remainder had some other qualification. In terms of marital status, 43.7% were married, 27.3% were single and not currently partners, 22.2% were partnered by not married, 5.4% were divorced, and the remainder were of another marital status.

### Measures

#### Belief in Conspiracy Theories Inventory

The version of the BCTI that we used was the 15-item, adapted version [15]. This version includes 14 items from the parent study [6] and an additional item added in a subsequent study [15]. The factor structure of this adapted version of the BCTI has not been previously investigated, but researchers have assumed that it retains its parent, one-factor structure. Internal consistency coefficients for this one-factor solution have tended to be acceptable (see Table 1). In the present study, all items were rated on a 9-point scale, ranging from 1 (*Completely false*) to 9 (*Completely true*). Higher scores on this scale reflect greater endorsement of a range of real-world conspiracy theories. BCTI items are reported in Table 4.

**Table 4:** Items and factor loadings for the Belief in Conspiracy Theory Inventory.

Item	Factor 1	Factor 2
8. The US government allowed the 9/11 attacks to take place so that it would have an excuse to achieve foreign (e.g., wars in Afghanistan and Iraq) and domestic (e.g., attacks on civil liberties) goals that had been determined prior to the attacks.	.81	-.06
5. The assassination of Martin Luther King, Jr., was the result of an organised conspiracy by US government agencies such as the CIA and FBI.	.78	-.07
4. US agencies intentionally created the AIDS epidemic and administered it to Black and gay men in the 1970s.	.77	.02
15. Government agencies in the UK are involved in the distribution of illegal drugs to ethnic minorities.	.76	-.26
3. The US government had foreknowledge about the Japanese attack on Pearl Harbour, but allowed the attack to take place so as to be able to enter the Second World War.	.71	-.19
11. Princess Diana’s death was not an accident, but rather an organised assassination by members of the British royal family who disliked her.	.61	.16
1. A powerful and secretive group, known as the New World Order, are planning to eventually rule the world through an autonomous world government, which would replace sovereign government.	.69	.14
2. SARS (Severe Acute Respiratory Syndrome) was produced under laboratory conditions as a biological weapon.	.67	.25
13. The Coca Cola company intentionally changed to an inferior formula with the intent of driving up demand for their classic product, later reintroducing it for their financial gain.	.66	-.12
9. The assassination of John F. Kennedy was not committed by the lone gunman, Lee Harvey Oswald, but was rather a detailed, organised conspiracy to kill the President.	.65	.25
6. The Apollo moon landings never happened and were staged in a Hollywood film studio.	.65	.17
12. The Oklahoma City bombers, Timothy McVeigh and Terry Nichols, did not act alone, but rather received assistance from neo-Nazi groups.	.64	-.01
14. Special interest groups are suppressing, or have suppressed in the past, technologies that could provide energy at reduced cost or reduced pollution output.	.62	-.08
7. Area 51 in Nevada, US, is a secretive military base that contains hidden alien spacecraft and/or alien bodies.	.57	.72
10. In July 1947, the US military recovered the wreckage of an alien craft from Roswell, New Mexico, and covered up the fact.	.60	.69

#### Conspiracy Mentality Questionnaire

Although there are 12- and 5-item version of the CMQ, we used the 5-item version of the scale because this is the more widely-used measure in the literature (see Table 2). Bruder et al. [21] reported that the 5-item CMQ had a one-dimensional structure using EFA and that the fit was adequate in German- and English-speaking samples using multi-group CFA. Although the response scale for this measure may be criticised for being difficult-to-understand, we maintained its original format in the present study. Participants were asked to respond on an 11-point scale ranging from 0% (*Certainly not*) to 100% (*Certain*). Higher scores on this scale reflect greater generic conspiracist ideation. CMQ items are reported in Table 5.

**Table 5:** Items and factor loadings for the Conspiracy Mentality Questionnaire.

Item	Factor1
5. I think that there are secret organizations that greatly influence political decisions.	.85
4. I think that events which superficially seem to lack a connection are often the result of secret activities.	.85
1. I think that many very important things happen in the world, which the public is never informed about.	.79
3. I think that government agencies closely monitor all citizens.	.77
2. I think that politicians usually do not tell us the true motives for their decisions.	.70

#### Generic Conspiracist Beliefs Scale

We used the 15-item version of the GCBS proposed [22]. The 15 items were selected by Brotherton and colleagues [22] from a larger pool of items to be representative of the five-factor solution reported in the parent study. The authors [22] reported that a five-factor solution had adequate fit using CFA and that this model also had better fit than a one-factor solution with all items. All subsequent studies have used total scores, rather than the five-factor solution, generally reporting acceptable internal consistency coefficients (see Table 2). In the present study, items were rated on a 5-point scale, ranging from 1 (*Definitely not true*) to 5 (*Definitely true*). Higher scores on this measure reflect greater generic conspiracist ideation. GCBS items are reported in Table 6.

**Table 6:** Items and factor loadings for the Generic Conspiracist Beliefs Scale. Values in bold indicate items that loaded onto a factor.

Item	Factor 1	Factor 2
3. The government uses people as patsies to hides its involvement in criminal activities.	**.79**	.20
15. A lot of important information is deliberately concealed from the public out of self-interest.	**.79**	.12
1. The government is involved in the murder of innocent citizens and/or well-known public figures, and keeps this a secret.	**.76**	.26
14. New and advanced technology which would harm current industry is being suppressed.	**.74**	.18
2. The government permits or perpetrates acts of terrorism on its own soil, disguising its involvement.	**.70**	.40
13. Groups of scientists manipulate, fabricate, or suppress evidence in order to deceive the public.	**.69**	.34
4. The power held by heads of state is second to that of small, unknown groups who really control world politics.	**.66**	**.45**
12. Experiments involving new drugs or technologies are routinely carried out on the public without their knowledge or consent.	**.66**	**.57**
6. Certain significant events have been the result of the activity of a small group who secretly manipulate world events.	**.62**	**.51**
10. The spread of certain viruses and/or diseases is the result of deliberate, concealed efforts of some organisations.	**.58**	**.57**
7. Secret organisations communicate with extraterrestrials, but keep this fact from the public.	.18	**.84**
9. Some UFO sightings and rumours are planned or staged in order to distract the public from real alien contact.	.23	**.83**
8. Evidence of alien contact is being kept from the public.	.21	**.81**
11. Technology with mind-control capacities is used on people without their knowledge.	.36	**.62**
5. A small, secret group of people is responsible for making all major world decisions, such as going to war.	**.55**	**.57**

#### One-Item Conspiracy Measure

We included the one-item conspiracy measure [18]. In this measure, participants are first presented with instructions that allude to some political and social events being debated. Participants are then asked to rate the following item: “I think that the official version of the events given by authorities very often hides the truth”. The item was rated on a 9-point scale, ranging from 1 (*Completely false*) to 9 (*Completely true*), so that higher scores reflect greater generic conspiracist ideation.

#### 9/11 conspiracy theories

As a measure of convergent validity, we included a subscale from the 9/11 Conspiracist Beliefs Scale [6]. The parent scale consisted of 17 items, but the authors [6] reported, using EFA, that the scale consists of two factors that measure general 9/11 conspiracist beliefs (10 items) and beliefs that the U.S. government conspired to cover-up what happened on September 11, 2011 (7 items). In the present study, only the former subscale was used, with items rated on a 9-point scale ranging from 1 (*Completely false*) to 9 (*Completely true*). To check that this subscale was indeed one-dimensional, we submitted the 10 items to principal-axis EFA using the total sample (*N* = 803). Bartlett’s test of sphericity, χ^2^(45) = 9453.29, *p* < .001, and the Kaiser-Meyer-Olkin (KMO) measure of sampling adequacy, KMO = .96, indicated that the 10 items had adequate common variance for factor analysis. An EFA with quartimax rotation revealed a single factor (λ = 7.90, variance explained = 79.0%), with all items having excellent loadings (≥ .81). An overall subscale score was, therefore, computed as the mean of the relevant 10 items, so that higher scores reflect greater endorsement of general 9/11 conspiracist beliefs. Swami et al. [6] reported that this subscale had acceptable internal consistency (Cronbach α = .95) and good patterns of construct and convergent validity. In the present study, Cronbach α for this scale was .97.

#### Anti-vaccination conspiracy theories

As a second measure of convergent validity, we included an 8-item measure of belief in anti-vaccination conspiracy theories [13]. All items were rated on a 7-point scale, ranging from 1 (*Strongly disagree*) to 7 (*Strongly agree*). In the parent study, the authors [13] reported that total scores on the scale had acceptable internal consistency (Cronbach α = .85), but neglected to examine the scale’s factor structure. We therefore subjected the 8 items to principal-axis EFA using the total sample. Bartlett’s test of sphericity, χ^2^(28) = 5306.46, *p* < .001, and the KMO measure of sampling adequacy, KMO = .90, indicated that the 8 items had adequate common variance for factor analysis. We initially computed a principal-axis EFA with quartimax rotation, but because the results indicated a multi-dimensional factor structure, we repeated the analysis using varimax rotation. The results indicated two factors with λ > 1.0 (3.37 and 2.87, respectively) and parallel analysis indicated that both factors should be extracted. Item loadings are reported in S1 Table. Four items loaded onto the primary factor, which tapped the belief that vaccinations are used as a population tracking mechanism (Cronbach α = .92, 42.1% of the variance explained). Four items loaded onto a secondary factor, tapping the belief that the dangers of vaccinations are being covered-up; however, two of these items also cross-loaded onto the primary factor, leaving two items in the secondary factor. Because Tabachnick and Fidell [29] do not recommend the use of subscales with less than three items, we elected to discard the secondary factor. The retained subscale included 4 items that tap the conspiracist belief that vaccinations are being used as population tracking mechanism (Cronbach α = .92).

#### Demographics

Participants provided their demographic details consisting of sex, age, current marital status, highest educational qualifications, and ethnicity.

### Statistical analyses

#### Exploratory factor analysis

We used a two-step procedure to examine the factor structures of the BCTI, GCBS, and CMQ. First, data from one-half of the sample (*n* = 402) was randomly selected via a computer-generated random seed. The factor structures of the three scales were then assessed using principal-axis EFA for this subsample using SPSS v.22. This method allowed us to test for the best-fitting model for our dataset, without *a priori* limitations in terms of modelling [30]. The sample size for all three scales met conservative 10:1 participant-to-item requirements for EFA [9]. Following standard guidelines [31], items were submitted to EFA if they passed standard criteria for item distribution (standardised kurtosis values > 10.0 suggest a problem), average correlation with the other items (items with *r* < .40 should be dropped), and item-total correlation (items should be dropped with corrected-item total correlations are < .30). For the BCTI and CMQ, we used quartimax rotations because of the expectation of a single, orthogonal factor; for the GCBS, we used a varimax rotation because we expected an inter-correlated, multidimensional model [32–33].

The number of factors to be extracted was determined by factor eigenvalues (λ) above 1.0 (the EGV1 criterion), examination of the scree-plot, and—where more than one factor was identified through rotation—the results of parallel analysis [34]. The latter was used because scree-plot inspection and the EGV1 criterion are known to lead to over-extraction of factors [35]. Parallel analysis works by creating random datasets with the same number of cases and variables as the actual dataset [36] Factors in the actual data are only retained if their eigenvalues are greater than the mean of eigenvalues from the random data [34]. Factor loadings were interpreted using Tabachnick and Fidell’s [29] recommendations (i.e., > .71 = excellent, > .63 = very good, > .55 = good, > .45 = fair, and > .32 = poor).

#### Confirmatory factor analysis

Data from the second split-half subsample (*n* = 401) was submitted for CFA using the Analysis of Moment Structures Program (AMOS v.23) [37]. Hypothesised modelling was based on the results of the earlier EFA, as well as hypothesised models from earlier studies where there were discrepancies. Standard goodness-of-fit indices were selected *a priori* to assess the measurement models. The normed model chi-square (χ²_normed_) is reported with lower values of the overall model χ² indicating goodness-of-fit. A χ²_normed_ value of < 3.00 indicates good fit [38]. The Steiger-Lind root mean square error of approximation (RMSEA) and its 90% confidence interval provide a correction for model complexity. RMSEA values close to .06 indicate a good fit, with values ranging to .10 representing a mediocre fit [38]. The standardised root mean square residual (SRMR) assesses the mean absolute correlation residual and is a badness-of-fit index: the smaller the SRMR, the better the model fit. A cut-off value for SRMR is recommended to be “close to” or < .09 [38]. The comparative fit index (CFI) measures the proportionate improvement in fit by comparing a target model with a more restricted, nested baseline model. The CFI reflects a goodness-of-fit index and is recommended to “close to” or > .95 for adequate fit [38]. Even so, these recommended cut-off values should be considered subjective guidelines [39–40]. We also examined standardised parameter estimates.

Factor loadings for CFA were interpreted using Comrey and Lee’s [41] recommendations (i.e., > .71 = excellent, > .63 = very good, > .55 = good, > .45 = fair, and > .32 = poor). The potential to improve the accuracy of each model was also evaluated through consultation of modification indices. Modification indices are estimates that identify potentially significant adjustments that could be made to the model (e.g., a covariance of the error terms for two indicators [42]. However, any modification to the existing model should make theoretical sense, rather than simply from analytical addition or subtraction of a parameter [43]. Further, the fit of the model cannot be improved by allowing an indicator to load onto another latent variable [44].

#### Supplemental analyses

For both subsamples, internal consistency coefficients were computed using Cronbach α. Although Nunnally [9] is widely interpreted as indicating that an internal consistency coefficient of .70 is acceptable, this is in fact a myth [45]. He, in fact, advocated a more conservative cut-off of .80, which we applied here. To assess convergent validity, we computed bivariate correlations between all included variables. According to Lipsey and Wilson [46], correlations of .10 are considered small, correlations of .25 are considered medium, and correlations of .40 are considered large.

## Results

### Belief in Conspiracy Theories Inventory

#### Exploratory factor analysis

The BCTI items were examined for normality of distribution and were found to be lower than limits, pre-empting transformation. The size of Kaiser-Meyer-Olkin (KMO) measure of sampling adequacy, KMO = .93, suggested that the BCTI items had adequate common variance for factor analysis, and Bartlett’s test of sphericity, χ^2^(105) = 3120.96, *p* < .001, indicated that the correlation matrix was factorable. The results of the EFA revealed two factors with λ > 1.0 (7.11 and 1.33). However, inspection of the scree-plot suggested one primary factor and a steep cut-off to the secondary factor. The results of parallel analysis showed that the mean of the first λ for the random data was smaller than the real data counterpart, whereas the mean of the second λ was larger than the second λ for the real data. These findings suggest that a single factor should be extracted. All 15 items had good loadings on this factor (see Table 4), which explained 47.4% of the total item variance. Cronbach α for the overall BCTI score, computed as the mean of all 15 items, was .92.

#### Confirmatory factor analysis

**Fig 1:**
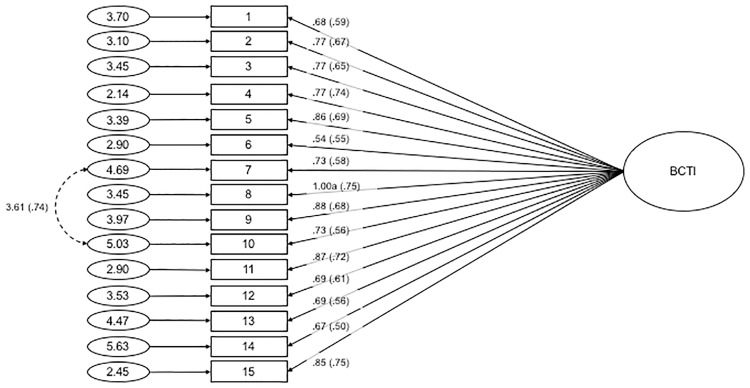
Path diagram and estimates for the Belief in Conspiracy Theories Inventory. Item numbers in the figure reflect the item number in Table 4. The large circle is the latent construct, with the rectangles representing measured variables, and the small circles with numbers are the residual variables (variances). The factor loadings are standardised in parenthesises, and the unstandarised values outside, with both being reported following the guidelines of Kline [42]. Significance levels were determined by critical ratios (all *p* < .001). The factor loadings were fixed at the indicated value (1.00a).

CFA was conducted on all 15 items of the BCTI, where all items loaded onto a single latent variable, indicated by the initial EFA of the split-half subsample. Fit indices values were found to be: χ²(90, *N* = 401) = 585.008, χ²_normed_ = 6.500, CFI = .824, RMSEA = .117 with 90% *CI* = .108-.126, SRMR = .063. Since the fit indices values of analysis were not found to be at acceptable intervals, suggested modification indices were taken into account to improve the model. Modification indices were consulted to free error covariances between items #7 and #10. The standardised estimates of factor loadings for this modified model were acceptable (see Fig 1 for the path diagram and standardised estimates). This one-dimensional structure provided an acceptable fit to the data: χ²(89, *N* = 401) = 278.638, χ²_normed_ = 3.131, CFI = .933, RMSEA = .073 with 90% *CI* = .063-.083, SRMR = .047. We were, therefore, able to compute an overall score as the mean of all 15 items. Cronbach α for the overall score was. .91.

### Conspiracy Mentality Questionnaire

#### Exploratory factor analysis

Tests of normality of distribution showed that the CMQ items were lower than limits. The size of KMO measure of sampling adequacy, KMO = .96, suggested that the BCTI items had adequate common variance for factor analysis, and Bartlett’s test of sphericity, χ^2^(10) = 956.54, *p* < .001, indicated that the correlation matrix was factorable. The results of the EFA revealed a single factor with λ = 3.15, which explained 63.0% of the variance. All 5 items had excellent loadings on this factor (see Table 5). Cronbach α for the overall CMQ score, computed as the mean of all 5 items, was .85.

#### Confirmatory factor analysis

**Fig 2:**
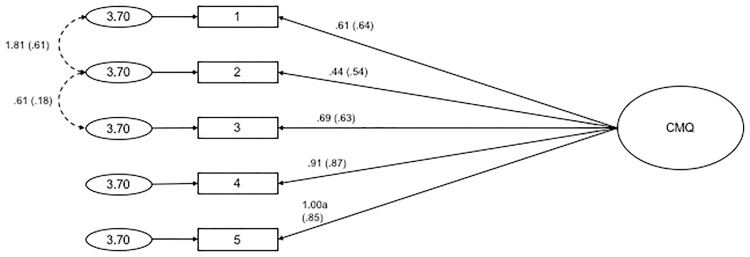
Path diagram and estimates for the Conspiracy Mentality Questionnaire. Item numbers in the figure reflect the item number in Table 5. The large circle is the latent construct, with the rectangles representing measured variables, and the small circles with numbers are the residual variables (variances). The factor loadings are standardised in parenthesises, and the unstandarised values outside, with both being reported following the guidelines of Kline [42]. Significance levels were determined by critical ratios (all *p* < .001). The factor loadings were fixed at the indicated value (1.00a).

CFA was conducted on the 5 items of the CMQ, where all items loaded onto a single latent variable. Fit indices values were found to be: χ²(5, *N* = 401) = 194.646, χ²_normed_ = 38.929, CFI = .812, RMSEA = .308 with 90% *CI* = .272-.346, SRMR = .081. Modification indices were consulted to free error covariances (items #1 and #2, and items #2 and #3). The standardised estimates of factor loadings for this model were acceptable (see Fig 2 for the path diagram and standardised estimates). This one-dimensional structure provided poor fit to the data: χ²(3, *N* = 401) = 22.859, χ²_normed_ = 7.620, CFI = .980, RMSEA = .129 with 90% *CI* = .083-.180, SRMR = .028. These results suggest that one-dimensional factor structure of the CMQ in this split-half subsample was problematic and did not achieve adequate fit indices. For this reason, we did not compute a total score for this split-half subsample.

### Generic Conspiracist Beliefs Scale

#### Exploratory factor analysis

The GCBS items were examined for normality of distribution and were found to be lower than limits. The size of the KMO (.94) and Bartlett’s test of sphericity, χ^2^(105) = 4292.37, *p* < .001, showed that the 15 GCBS items had adequate common variance for EFA. The results of the EFA revealed only two factors with λ > 1.0 (5.50 and 4.19) and the scree-plot showed a steep cut-off between the primary and secondary factors. However, the results of parallel analysis indicated that both factors should be extracted: the mean of the first and second λ for the random data were smaller than the real data counterparts. Eleven items loaded onto the first factor and 10 items loaded onto the second factor, but of these 5 items cross-loaded onto both factors (see Table 6). Tabachnick and Fidell [29] recommended that all cross-loading items should be eliminated, leaving 6 items for the first factor (Cronbach α = .89) and 4 for the second (Cronbach α = .85). Because of the diversity of items that loaded onto the first factor, we termed this factor General Conspiracist Beliefs. Three of the four items that loaded on the second factor related to extraterrestrial beliefs, so we termed this factor Extraterrestrial Conspiracist Beliefs.

#### Confirmatory factor analysis

**Fig 3:**
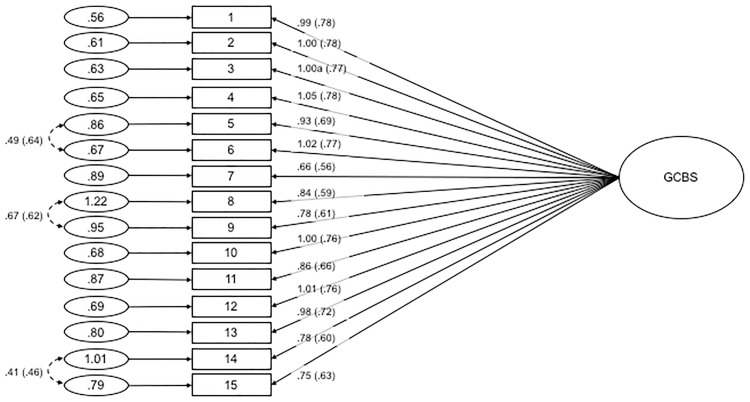
Path diagram and estimates for the one-factor Generic Conspiracist Beliefs Scale. Item numbers in the figure reflect the item number in Table 6. Item numbers in the figure reflect the item number in Table 6. The large circle is the latent construct, with the rectangles representing measured variables, and the small circles with numbers are the residual variables (variances). The factor loadings are standardised in parenthesises, and the unstandarised values outside, with both being reported following the guidelines of Kline (2011). Significance levels were determined by critical ratios (all *p* < .001). The factor loadings were fixed at the indicated value (1.00a).

Using CFA, we tested three separate models for the GCBS: a one-factor model where all 15 items loaded onto a single latent variable, a five-factor model where the 15 items loaded onto the five factors as per the parent study [22], and a 10-item, two-factor model as indicated by the initial EFA of the split-half subsample. Fit indices for the one-factor model were found to be poor: χ²(90, *N* = 401) = 1122.934, χ²_normed_ = 12.477, CFI = .751, RMSEA = .169 with 90% *CI* = .161-.178, SRMR = .089. Modification indices were consulted to free error covariances (items #5 and #6, #8 and #9, and #14 and #15). However, the fit of this one-dimensional structure remained poor: χ²(87, *N* = 401) = 652.389, χ²_normed_ = 7.499, CFI = .864, RMSEA = .127 with 90% *CI* = .118-.137, SRMR = .074 (see Fig 3 for standardised estimates of factor loadings). Based on these results, we concluded that there is little support for a one-dimensional structure of the GBCS.

**Fig 4:**
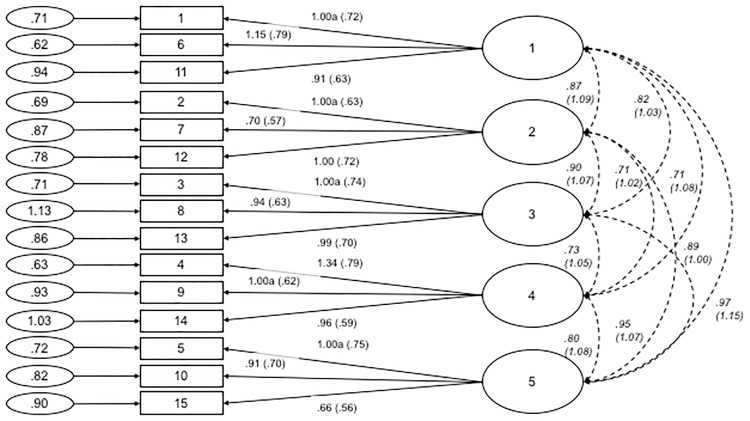
Path diagram and estimates for the five-factor Generic Conspiracist Beliefs Scale. Item numbers in the figure reflect the item number in Table 6. The large circles are the latent construct, with the rectangles representing measured variables, and the small circles with numbers are the residual variables (variances). The factor loadings are standardised in parenthesises, and the unstandarised values outside, with both being reported following the guidelines of Kline [42]. Significance levels were determined by critical ratios (all *p* < .001). Estimates of covariance between exogenous variables are displayed in italics. The factor loadings were fixed at the indicated value (1.00a).

Next, we examined the fit of the 15-item, five-factor model proposed by Brotherton et al. (2013). The fit indices for this structure were also found to be poor: χ²(80, *N* = 401) = 1038.819, χ²_normed_ = 12.985, CFI = .769, RMSEA = .173 with 90% *CI* = .164-.183, SRMR = .090. Modification indices were not consulted to adjust the model as all modifications would lead to indicators being loaded onto another latent variable or have little theoretical sense. Fig 4 depicts the factor structure and standardised estimates of factor loadings for the five-factor model. Based on these results, we concluded that the five-factor model should be discarded.

**Fig 5:**
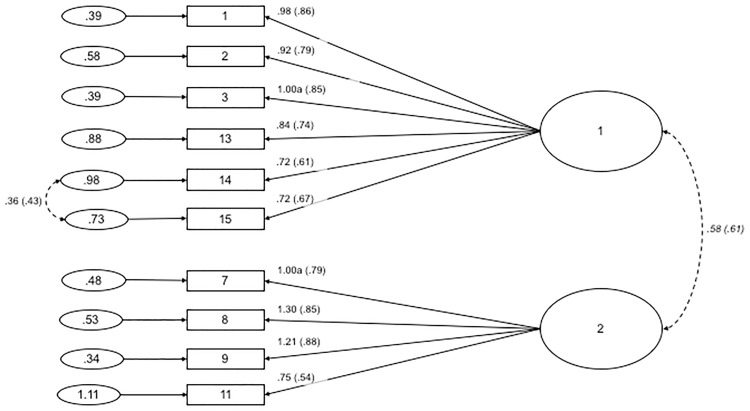
Path diagram and estimates for the two-factor Generic Conspiracist Beliefs Scale. Item numbers in the figure reflect the item number in Table 6. The large circles are the latent construct, with the rectangles representing measured variables, and the small circles with numbers are the residual variables (variances). The factor loadings are standardised in parenthesises, and the unstandarised values outside, with both being reported following the guidelines of Kline [42]. Significance levels were determined by critical ratios (all *p* < .001). Estimates of covariance between exogenous variables are displayed in italics. The factor loadings were fixed at the indicated value (1.00a).

Finally, the 10 items that were retained in a two-factor structure from the first split-half subsample was analysed. This model was also found to have poor fit: χ²(34, *N* = 401) = 261.125, χ²_normed_ = 7.680, CFI = .902, RMSEA = .129 with 90% *CI* = .115-.144, SRMR = .080. Modification indices were consulted to free error covariances between items under the same factor between items #14 and #15. The standardised estimates of factor loadings for this model were acceptable (see Fig 5 for the path diagram and standardised estimates). The resulting two-factor structure was more acceptable in terms of fit, though still problematic on a number of indices: χ²(33, *N* = 401) = 191.556, χ²_normed_ = 5.805, CFI = .931, RMSEA = .110 with 90% *CI* = .095-.125, SRMR = .076. These results suggest that, even following modifications, the two-factor model of the GCBS continued to have poor fit and so was omitted from further analyses.

### Convergent validity

Descriptive statistics for all variables are reported in Table 7. We computed inter-scale, bivariate correlations between all variables for the EFA and CFA split-halves separately. As can be seen in Table 7, there were significant inter-correlations between all variables for the EFA split-half, but evidence of convergent validity was strongest for the BCTI and weakest for the CMQ and one-item conspiracy measure. There were also significant inter-correlations between all variables for the CFA-split half. However, evidence of convergent validity was strongest for the BCTI and weakest for the one-item conspiracy measure.

**Table 7:** Bivariate Correlations between all measures included in the present study (EFA Split-Half Subsample in the Top Diagonal, CFA Split-Half Subsample in the Bottom Diagonal).

	(1)	(2)	(3)	(4)	(5)	(6)	(7)	*M*	*SD*
(1) Belief in Conspiracy Theories Inventory		.64	.75	.80	.71	.78	.65	5.57	1.85
(2) Conspiracy Mentality Questionnaire	.71		.75	.55	.68	.53	.33	7.55	1.86
(3) GBCS—General Beliefs	-	-		.60	.74	.64	.40	3.21	0.99
(4) GCBS—Extraterrestrial Beliefs	.72	-	-		.58	.66	.58	2.28	1.07
(5) One-Item Conspiracy Measure	.71	-	-	-		.40	.36	5.68	2.31
(6) 9/11 Conspiracist Beliefs	.75	-	-	-	.32		.68	2.95	2.24
(7) Anti-Vaccination Beliefs	.53	-	-	-	.24	.66		1.82	1.34
*M*	3.62	-	-	-	5.40	2.96	1.72		
*SD*	1.76	-	-	-	2.43	2.18	1.00		

*Note*. All *p* < .001

## Discussion

In this study, we examined the factorial and convergent validity of four different scales used to measure conspiracist ideation. In very broad outline, our results highlight concerns with the ways in which conspiracist ideation is currently operationalised and measured in the future. Here, we begin by discussing our findings in relation to each of the four included measures, before turning our attention to the future.

### Belief in Conspiracy Theories Inventory

Of the four measures we included in the present work, the BCTI showed the strongest evidence of factorial validity. Using EFA, we found support for the idea that the scale reduces to a single dimension, onto which all items load adequately. In addition, using CFA, we found that the one-dimensional model of the BCTI had acceptable fit and, in both sub-samples, overall scores had acceptable internal consistency. This is perhaps not surprising when we consider that belief in conspiracy theories is thought to be monological; that is, endorsement of one conspiracy theory makes acceptance of other conspiracy theories more likely [6, 8, 26]. In addition, of the four scales, the BCTI showed the strongest correlations with measures used to establish convergent validity. Of course, the main limitation of the BCTI is a conceptual one: it is uncertain to what extent the measure truly taps conspiracist ideation, as opposed to endorsement of a range of conspiracy theories (in the present study, correlations with generic measures of conspiracist ideation were generally strong). It is also unclear to what extent the BCTI will remain temporally stable in the long-term, as individual items may become obsolete, or to what extent individual items will be cross-culturally relevant.

### Conspiracy Mentality Questionnaire

In our EFA, we found that the 5 items of the CMQ reduced to a single dimension with acceptable internal consistency. However, using CFA, we found that the one-dimensional model had poor fit, even following modifications. Moreover, in the EFA sub-sample, evidence of convergent validity was moderate at best. We believe the poor factorial and convergent validity of the CMQ may reflect underlying problems with the construct validity of this measure. Specifically, we suggest that some items of the CMQ may not tap conspiracist ideation, but may reflect rational beliefs about the current state of the world. For example, given current knowledge, item #3 (“I think that government agencies closely monitor all citizens”) could be construed as factual and requires no conspiracist mentality. The high mean scores for this measure (well above the scale mid-point) suggest that participants in this study were indeed rating some items of the CMQ as factually correct. In short, we suggest that there may be underlying problems with the construct validity of the CMQ, which affects its latent dimensionality.

### Generic Conspiracist Beliefs Scale

In the parent study [22], the GCBS developers suggested that this scale consists of five factors and that the five-factor model had better fit than overall scores. In the present work, we failed to find support for either of these models using both EFA and CFA. Instead, our EFA suggested a truncated, two-factor model should be extracted. However, our CFA suggested that none of the models of the GCBS had adequate fit even following modifications, suggestive of inherent problems with the dimensionality of this measure. It is possible that part of the problem with this measure is confusion about its latent structure and whether it taps a single or multiple dimensions of conspiracist ideation (see below). At best, the GCBS may tap different dimensions of conspiracist ideation (e.g., general conspiracist ideation versus conspiracist beliefs about extraterrestrial life); at worst, it may tap multiple dimensions that do not cohere very well. Overall, the present findings raise concerns about the use of this measure.

### One-Item Conspiracy Measure

We were unable to assess the factorial validity of the measure developed by Lantian et al. [18], given its single-item nature. However, our assessment of its convergent validity returned less than ideal results. This was to be expected given the inherently low reliability of any one-item measure, regardless of its content and purpose. Of the four measures included here, correlations between the one-item conspiracy measure and indices of convergent validity were the weakest.

### Looking to the future

One general conclusion that might be drawn on the basis of the present dataset is that, while endorsement of a range of conspiracy theories is indeed monological, there are problems with the measurement of conspiracist ideation in scales currently in use. It is possible that the CMQ and GCBS are modelling a substantial amount of noise (e.g., measurement error, sampling fluctuations) and that latent factor structures depend on arbitrary properties in the data. As a result, the uncovered factor structures associated with these scales may differ between studies (as in the case between our dataset and earlier studies) or within studies (as in the differences between our EFA and CFA subsamples). The most straightforward solution here is that, where scholars use the CMQ or GCBS, they should examine the factor structures of these scales in their dataset rather than assuming these measures are one-dimensional.

Beyond this general point, we do not recommend the use of the CMQ and the one-item conspiracy measure in future studies. The CMQ appears to have poor factorial validity (and likely poor construct validity), whereas the one-item conspiracy measure appears to have weak convergent validity. Scholars who wish to measure generic conspiracist ideation may find it better to use the GCBS, but they should pay careful attention to (and report) its factor structure within studies. Our findings suggest the possibility that conspiracist ideation may be multi-dimensional and, as a result, scholars should not assume that the GBCS—or other measures of conspiracist ideation—are necessarily one-dimensional. More broadly, we suggest that there may utility in returning to a more careful consideration of theory, especially the conceptualisation of conspiracist ideation as a latent trait.

One way to resolve these issues would be to return to the 75 items developed by Brotherton et al. [22], and submit these items to exploratory factor analysis using a suitably large sample (i.e., a sample of no less than 750 individuals [9]. Doing so may highlight alternative factor structures that could then be re-assessed using CFA. Another way forward would be to subject all the items from conceptually similar scales (e.g., the CMQ and GCBS) to a single factor analysis, to examine the extent of conceptual overlap and possible item redundancy. In addition, future studies may also wish to revisit the factorial validity of the Conspiracy Theory Belief Scale, which we omitted in the present work. More broadly, we strongly recommend that all scholars working on conspiracist ideation pay closer attention to issues of measurement. Our data suggest that insufficient attention has been given to factorial and convergent validity, and that this may have introduced a degree of measurement bias into previously-reported findings. Indeed, it was concerning that even one of our measures of convergent validity [13] had not been submitted to factor analysis and that we found a divergent structure to what the authors of the parent study had assumed. Looking ahead, we highlight the need for improved assessments of the psychometric properties of scales used to measure conspiracist ideation. At a minimum, we recommend the reporting of factor analytic findings for all measures, particularly novel scales.

### Limitations and conclusion

One might argue that our findings are less reliable, for whatever reason, compared to the parents studies reported in Tables 1–3. Even were this the case, however, our findings highlight discrepancies in factorial validity that cannot, and should not, be overlooked. Indeed, our study benefits from a relatively large, culturally homogeneous sample and a two-step assessment of factorial validity. Nevertheless, there were limitations to the present study, too. First, our use of an online sample means that we cannot be certain about the generalisability of our findings. In particular, the present results may be geographically and culturally limited. In addition, we did not assess the temporal stability of the four measures, and this could be usefully (re)examined in future studies. Including alternative, and a broader range of, measures of convergent validity would also be a welcome addition in future studies.

In addition, because our sample was self-selecting, the possibility of sampling biases should be considered (e.g., those who were most interested in the topic may have been more likely to participate and complete the survey without dropping-out). Likewise, the inclusion of idiosyncratic instructions typically included with each measure may have also led to biased results, and this is something that future studies should consider. These limitations notwithstanding, the present findings suggest that scholars working on conspiracist ideation need to pay more detailed attention to measurement issues in their studies. If the findings from this field of research are to be taken seriously, measurement issues need to be thoughtfully considered and dealt with by scholars. For now, we suggest that scholars wishing to measure conspiracist ideation may need to return to the drawing board.

## Supporting information

S1 File Syntax for the Belief in Conspiracy Theories Inventory. (SPS)

S2 File Syntax for the Conspiracy Mentality Questionnaire. (SPS)

S3 File Syntax for the Generic Conspiracist Beliefs Scale. (SPS)

S1 Table Items and factor loadings for the anti-vaccination conspiracy theories measure. Values in bold indicate items that loaded onto a factor.
(DOCX)
