# Correction: An Examination of the Factorial and Convergent Validity of Four Measures of Conspiracist Ideation, with Recommendations for Researchers

**DOI:** 10.1371/journal.pone.0336450

**Published:** 2025-11-11

**Authors:** Viren Swami, David Barron, Laura Weis, Martin Voracek, Stefan Stieger, Adrian Furnham

After publication of this article [1], the authors discovered that due to an error in data processing, the reported fit of the 5-factor Generic Conspiracist Beliefs Scale (GBCS) model was incorrect at time of original publication.

With this Correction, the authors report the corrected statistics and path diagram as initially appeared in Fig 4. See the authors’ description of these changes below:

“Using the correct item enumerators (matching those reported in Table 6), the fit statistics for the 5-factor model are: χ²(80, *N* = 401) = 255.611, χ²_normed_ = 3.195, CFI =.958, RMSEA =.074 with 90% *CI* =.064-.084, SRMR =.048 (see Figure 1).” Please see the corrected Fig 4 here.

**Fig 4:**
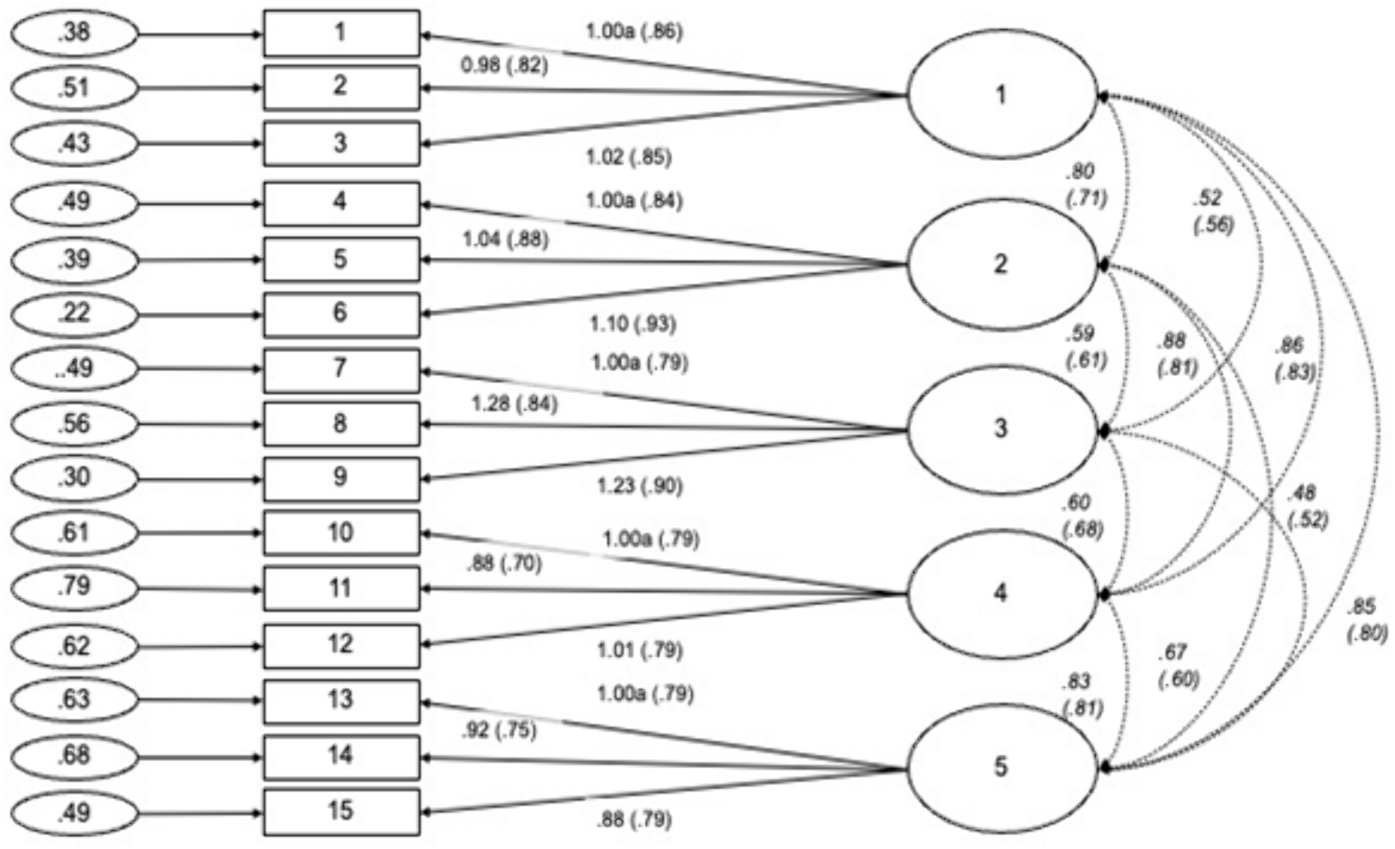
Path diagram and estimates for the five-factor Generic Conspiracist Beliefs Scale. Item numbers in the figure reflect the item number in Table 6. The large circles are the latent construct, with the rectangles representing measured variables, and the small circles with numbers are the residual variables (variances). The factor loadings are standardised in parenthesises, and the unstandarised values outside, with both being reported following the guidelines of Kline [42]. Significance levels were determined by critical ratios (all *p* < .001). Estimates of covariance between exogenous variables are displayed in italics. The factor loadings were fixed at the indicated value (1.00a).

Please see Table 8 here showing the previously unreported total-sample bivariate correlations among the 5-factor scores of the GCBS with 9/11 conspiracist beliefs and anti-vaccination beliefs.

**Table 8:** Bivariate correlations among the 5-factor scores of the GCBS (total sample).

	(1)	(2)	(3)	(4)	(5)	(6)
(1) GCBS 1: Government malfeasance	–					
(2) GCBS 2: Malevolent global conspiracies	.67	–				
(3) GCBS 3: Extraterrestrial cover-up	.52	.56	–			
(4) GCBS4: Personal well-being	.71	.72	.63	–		
(5) GCBS5: Control of information	.70	.58	.47	.67	–	
(6) 9/11 conspiracist beliefs	.63	.68	.61	.70	.47	–
(7) Anti-vaccination beliefs	.36	.48	.51	.55	.26	.65

The 5-factor model of GBCS scores in our second split-half subsample may have better fit than we previously indicated. However, given support elsewhere (and in our first split-half subsample) for a 2-factor model [2], a 3-factor model [3], a bi-factor model [4], as well as for a correlated 5-factor model [5] of GCBS scores, it appears that the observed dimensionality of this instrument lacks consensus, is unstable (if not erratic), thus suggesting that it may well depend on: (i) sampling frame and sample characteristics, (ii) analytic approach, (iii) survey language, (iv) recent time trends (increases) in the prevalence (endorsement rate) of conspiracy beliefs in the population, or (v) any combination thereof. Accordingly, we repeat the advice from our original report: “Scholars who wish to measure generic conspiracist ideation may find it better to use the GCBS, but they should pay careful attention to (and report) its factor structure within studies. Our findings suggest the possibility that conspiracist ideation may be multi-dimensional and, as a result, scholars should not assume that the GBCS – or other measures of conspiracist ideation – are necessarily one-dimensional.”
