# Supplementary material for: An examination of the factorial and convergent validity of four measures of conspiracist ideation, with recommendations for researchers
Source: PLoS One. 2017 Feb 23;12(2):e0172617. doi: 10.1371/journal.pone.0172617 (PMC5322923; doi:10.1371/journal.pone.0172617)
Supplement: S1 Table — Values in bold indicate items that loaded onto a factor. (DOCX) [file pone.0172617.s004.docx]

| Item | Factor 1 | Factor 2 |
| --- | --- | --- |
| 8. The flu vaccine allows the government to monitor the elderly through the implantation of tiny tracking devices. | **.92** | .24 |
| 7. Tiny devices are implanted in vaccines for use in mind control experiments. | **.91** | .22 |
| 3. Tiny devices are placed in vaccines to track people. | **.88** | .30 |
| 1. Immunizations allow governments to track and control people. | **.71** | .31 |
| 6. The government is trying to cover up the link between vaccines and autism. | .33 | **.82** |
| 4. Pharmaceutical companies, scientists and academics work together to cover up the dangers of vaccines. | **.40** | **.81** |
| 2. Vaccines are harmful, and this fact is covered up. | **.41** | **.81** |
| 5. Vaccines are not tampered with. | **-**.07 | **-.71** |
